# Can slowing the rate of water temperature decline be utilized to reduce the impacts of cold water pollution from dam releases on fish physiology and performance?

**DOI:** 10.1111/jfb.15002

**Published:** 2022-02-05

**Authors:** Monique A. Parisi, Craig E. Franklin, Rebecca L. Cramp

**Affiliations:** ^1^ School of Biological Sciences The University of Queensland Brisbane Queensland Australia

**Keywords:** conservation physiology, metabolic rate, phenotypic plasticity, swimming performance, thermal pollution

## Abstract

Cold water pollution (CWP) is caused by releases of unseasonably cold water from large, thermally stratified dams. Rapid and prolonged decreases in water temperature can have depressive effects on the metabolism, growth and swimming performance of fish. However, it is unknown if reducing the rate of temperature decrease could mitigate these negative effects by allowing thermal acclimation/acclimatization to occur. This study investigated the rate of temperature decrease as a potential CWP mitigation strategy in juvenile Murray cod *Maccullochella peelii*. *M. peelii* were exposed to a gradual, intermediate or rapid temperature decrease from 24 to 14°C. Energetic costs, locomotor performance, growth and survival were measured to determine if the initial thermal regime affected the thermal acclimation capacity of *M. peelii*. Cold exposure had significant acute and lasting depressive effects regardless of the rate of temperature decrease, although *M. peelii* showed varying degrees of thermal compensation in swimming performance and metabolism after 8 weeks of exposure to low temperatures. The short‐term effects of CWP‐like reductions in temperature are significant, but over time *M. peelii* can offset some of the depressive effects of CWP through thermal plasticity. This study highlights the importance of understanding physiological responses of fish to inform management and conservation. We conclude that rate of water temperature decline cannot be used to mitigate the sublethal effects of CWP on juvenile *M. peelii* but may still be useful for managing the negative effects in other native Australian fish species.

## 1 INTRODUCTION

Globally, freshwater ecosystems are heavily impacted by anthropogenic alterations (Grill *et al*., 2019; Vörösmarty *et al*., 2010). They are particularly threatened by overexploitation, pollution, flow modification, habitat degradation and invasive species (Arthington *et al*., 2010; Gatti, 2016). As a result, freshwater ecosystems are increasingly vulnerable to species losses (Grill *et al*., 2019). The regulation of waterways and construction of dams, weirs and culverts have created physical, thermal and hydrodynamic barriers to fish movement (Grill *et al*., 2019). In one of the largest freshwater systems in the world, Australia's Murray‐Darling Basin (MDB), barriers to fish movement have contributed to a 90% decline in native fish numbers and significant changes to species composition (Boys *et al*., 2009). In addition to physically restricting fish movements, regulation of water flows and the release of water from large dam impoundments can significantly alter the thermal regime of downstream waters (Boys *et al*., 2009). Cold water pollution (CWP) is a particular issue in the MDB, and occurs in the summer months when cold water is released from the bottom of large, thermally stratified impoundments (Boys *et al*., 2009; Lugg & Copeland, 2014; Preece, 2004). CWP can result in an abrupt drop in downstream water temperatures of up to 16°C, which may extend as far as 350 km downstream of the dam and can persist for many months (Lugg & Copeland, 2014; Preece, 2004).

For aquatic ectotherms such as fish, body temperature is dictated by water temperature and, through its impact on physiological processes, underpins key survival behaviours including foraging, predatory escape (Flore & Keckeis, 1998) and reproductive success (Kopf *et al*., 2014). For most fishes, physiological performance is optimized over a relatively narrow range of environmental temperatures that reflect the typical environmental temperatures experienced by the organism; exposure to temperatures either side of this optimal range can reduce physiological performance (Bennett, 1990). Acute exposure of fish to low water temperatures, such as during CWP events, can result in decreased swimming performance, growth and survival (Astles *et al*., 2003; Parisi *et al*., 2020). Recently, we showed that juvenile silver perch *Bidyanus bidyanus* (Mitchell, 1838) exposed to a rapid (6 h) 10°C decrease in temperature to mimic downstream conditions during a cold water release from a deep dam impoundment experienced both acute and chronic depressive effects of low temperatures on swimming performance and metabolic rate (Parisi *et al*., 2020). While it was clear that the magnitude of the temperature decrease was challenging for *B. bidyanus*, it is unknown if the impact of CWP‐like decreases in temperature could be ameliorated by reducing the rate at which water temperature declines. If so, this adjustment in temperature decline rate could potentially be used as a management strategy in cold water polluted reaches.

CWP is not the only thermal challenge for fish in freshwater ecosystems; fish contend with natural (seasonal) changes in water temperature by using a range of strategies, which may include moving to more suitable environments or adjusting the thermal sensitivity of physiological rate processes (via thermal acclimatization) to optimize performance at different temperatures (Seebacher *et al*., 2015). Thermal phenotypic plasticity allows fish to occupy environments with distinctly different seasonal temperatures and can buffer them against this thermal variability by reducing the extent to which low temperatures impair performance (*i.e*., thermal compensation *via* acclimatization) (Guderley, 2004; Wilson & Franklin, 2002). However, thermal compensation can be influenced by a number of factors, such as body size, exposure period (Rohr *et al*., 2018), thermal variability (Schulte *et al*., 2011) and rate of temperature change (Nilsson‐Örtman & Johansson, 2017). Rate of temperature change is particularly important for measurements of critical thermal tolerances (Beitinger *et al*., 2000), and affects growth (Eldridge *et al*., 2015) and stress responses (Tanck *et al*., 2000) in various fishes. Furthermore, the capacity of fish to compensate performance in response to extended exposure to low temperatures can also vary among species, life‐history stages and magnitude of temperature change (Donaldson *et al*., 2008). Although many fish species compensate physiological performance in response to seasonal reductions in water temperature, if/how fish use this strategy in response to anthropogenic changes in water temperature associated with instream barriers (*i.e*., CWP) is not as well understood.

The aim of this study was to investigate how the rate of water temperature change affects the thermal plasticity of the metabolic rate and swimming performance of freshwater fish and its implication for the management of water release strategies from thermally stratified dams. We measured the swimming performance (critical [*U*_crit_] and sprint [*U*_sprint_]) of juvenile Murray cod *Maccullochella peelii* (Mitchell 1838) at intervals over an 8‐week chronic exposure period following a series of graded temperature transitions from 24 to 14°C. We also measured the thermal compensation of swimming performance and metabolic rate following the 8‐week exposure period. We hypothesised that fish exposed to a more gradual rate of thermal reduction would experience less severe depressive effects on swimming performance compared to fish rapidly exposed to a 10°C reduction in water temperature. Similarly, following chronic exposure to low temperature, fish that underwent a slower rate of initial temperature decrease would have higher physiological performance than fish that were exposed to an intermediate or rapid decrease in water temperature.

## 2 MATERIALS AND METHODS

### 2.1 Study species

*M. peelii* are an Australian fish species native to the MDB. *M. peelii* populations have suffered significant declines due to anthropogenic activities and in some areas *M. peelii* have completely disappeared from former habitats (Koehn, 2015; Lintermans, 2007; Todd *et al*., 2005). Consequently, *M. peelii* are listed as “Vulnerable” under the Australian Federal Government's Environmental Protection and Biodiversity Conservation Act 1999. Specifically, barriers to fish movement, including CWP, are one of the leading factors contributing to this species' decline in the MDB (Boys *et al*., 2009).

### 2.2 Study design

Juvenile *M. peelii* (*n* = 88, 5–15 cm total length) were purchased from a commercial hatchery (Narrabri Fish Farm, New South Wales, Australia) and housed in two 1000 l recirculating aquarium systems with carbon‐filtered Brisbane tap water. Water changes occurred consistently *via* a drip feed system to ensure that water quality was maintained throughout the experiment. Temperature was maintained with heater/chiller units (TK1000; Teco, Ravenna, Italy) and monitored with Thermochron iButtons (iButtonLink, Wisconsin, USA). Fish were fed commercial fish food pellets and/or bloodworms to satiation daily. The photoperiod was set to a 12 h light and 12 h dark cycle. Before experiments began, fish were tagged with visible implant elastomer tags (Northwest Marine Technology, Washington, USA) to identify individuals. Fish were given 7 days to recover from tagging.

**FIGURE 1:**
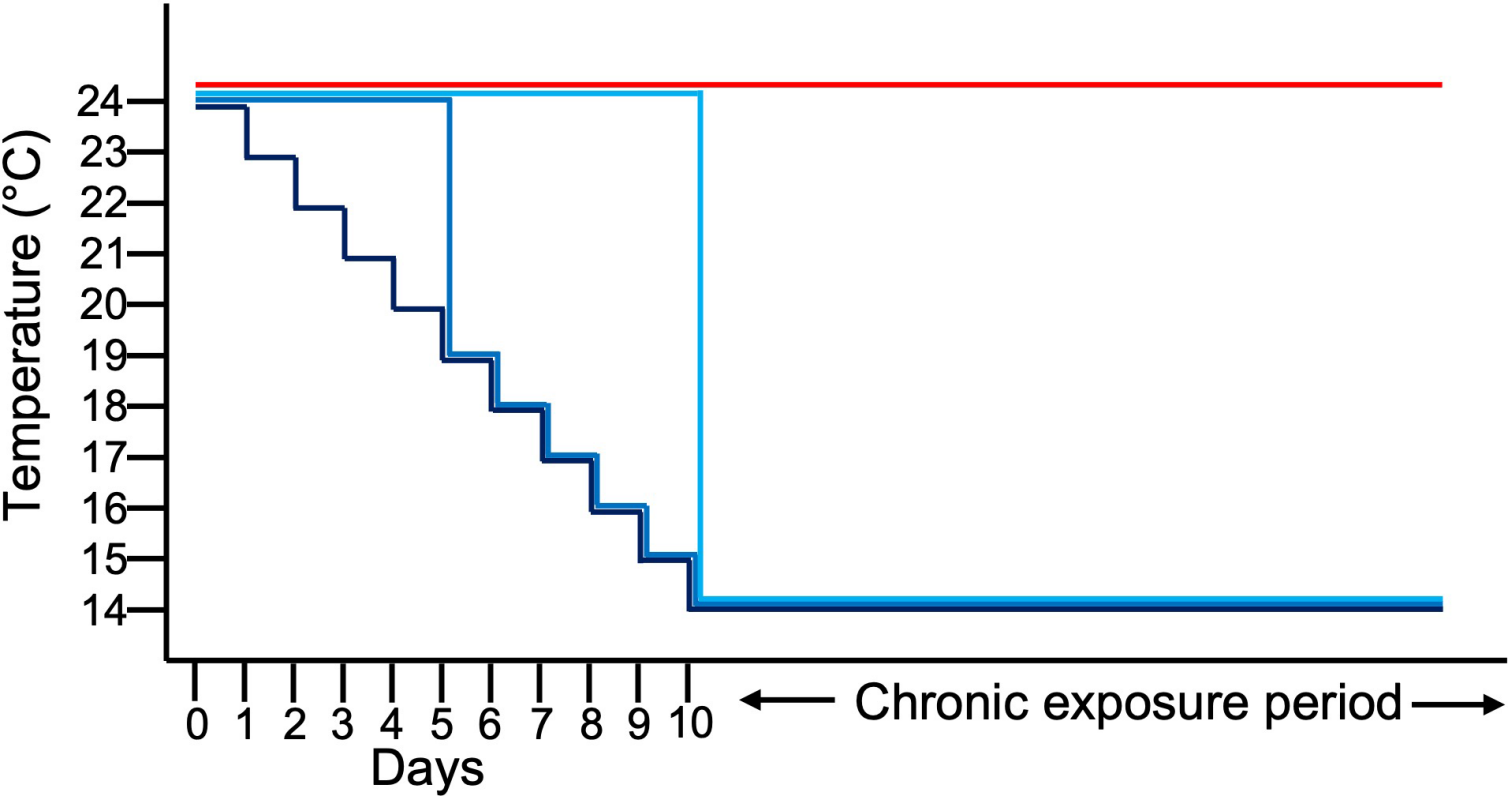
Schematic showing the initial rate of water temperature decrease for each treatment in the experiment. The ‘gradual’ treatment underwent a 1°C a day decrease in water temperature over 10 days until the water temperature reached 14°C. The ‘intermediate’ treatment underwent a 5°C decrease in water temperature in 1 day, followed by a 1°C a day decrease in water temperature for the following 5 days until the water temperature reached 14°C. The ‘rapid’ treatment underwent a 10°C decrease in water temperature in 1 day. The ‘control’ remained at 24°C throughout the entire experiment. (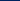) gradual; (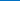) intermediate; (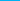) rapid; (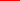) control

After tagging, fish were randomly selected for each treatment, with an even representation of size classes among treatments. Each treatment was allocated three 40 l tanks and within each treatment individuals were allocated to a tank by size to minimize aggression. Fish were stocked at a density of five to 10 individuals per tank. Water temperature was maintained at 24°C for the ‘warm’ treatment (*n* = 22) throughout the experiment. There were three different ‘cold’ treatments maintained at 14°C throughout the experiment, but the rate of temperature decrease for the initial exposure from 24 to 14°C differed between each cold treatment (Figure 1). The ‘gradual’ cold treatment (*n* = 22) was exposed to a 1°C a day decrease in water temperature over 10 days until the temperature reached 14°C. The ‘intermediate’ cold treatment (*n* = 22) was exposed to a 5°C drop in water temperature in 1 day, followed by a 1°C a day decrease in temperature for the following 5 days until the water temperature reached 14°C. The ‘rapid’ cold treatment (*n* = 21) was exposed to a 10°C decrease in water temperature in 1 day. The rapid treatment was chosen to mimic a cold water release from a dam (Astles *et al*., 2003). The chronic exposure period began once all cold exposure treatments had been at 14°C for 24 h. Animals were then maintained at this temperature for 8 weeks.

Swimming performance and growth were measured at each animal's treatment temperature at weeks 0, 2, 5 and 8 of the chronic exposure period. At week 9, the three cold treatments (at 14°C) were tested acutely (after 1 h) at the warm treatment temperature (24°C) and the warm treatment was tested acutely at 14°C (after 1 h). From weeks 10 to 14, metabolic rate measurements were recorded at the treatment temperature and the opposing temperature. Survival was recorded daily throughout the experiment. All fish were fasted for 24 h before swimming performance or metabolic rate measurements.

All experiments were conducted in accordance with the Australian Animal Care guidelines and approved by the University of Queensland animal ethics committee (Ethics Approval No. SBS/339/17).

### 2.3 Growth

Growth, measured as body mass (g) and total length (cm), was recorded for all fish after the completion of swimming performance and metabolic rate measurements.

### 2.4 Swimming performance

Swimming performance measurements were conducted in a thermostatically controlled 185 l recirculating swimming flume (Loligo, Tjele, Denmark; swimming chamber dimensions = L × W × H, 87.5 × 25 × 25 cm). Water temperature was controlled using a chiller (Hailea HC‐1000A, Guangdong, China) or submersible aquarium heating rods (Aqua Zonic, Singapore). Critical swimming speed (*U*_crit_) was measured following Brett (1964) and Rodgers *et al*. (2014). Fish were netted and placed into the swimming chamber for a settling period of at least 15 min prior to the swimming trial, which was enough time to allow fish to regain normal behaviour and adjust to the swim chamber (Kern *et al*., 2018). Water velocity was set at 10 cm s^−1^ for 5 min and then increased by 5 cm s^−1^ every 5 min until the fish fatigued (defined as the fish resting against the back wall of the swim chamber for 3 s). Fish would likely utilize both aerobic and anaerobic pathways during this *U*_crit_ protocol. Sprint swimming speed (*U*_sprint_) was measured following the protocol described by Starrs *et al*. (2011). After the 15 min settling period, water velocity was increased to 10 cm s^−1^ for 5 min. Water velocity was then increased by 5 cm s^−1^ every 10 s until the fish fatigued. *U*_crit_ and *U*_sprint_ were calculated using the equation from Brett (1964):

$$U_{\text{crit}}\;\text{or}\;U_{\text{sprint}}=U_{f}+\left[U_{i}\left(T_{f}/T_{i}\right)\right]$$

where *U*_f_ is the highest sustained water velocity achieved by the fish (cm s^−1^), *U*_i_ is the water velocity increment (5 cm s^−1^), *T*_f_ is time swam during the final increment and *T*_i_ is the entire velocity interval (*U*_crit_ 300 s or *U*_sprint_ 10 s).

### 2.5 Metabolic rate

Oxygen uptake $\left(\dot{M}O_{2}\right)$ was measured using intermittent‐flow respirometry to determine the routine and maximum metabolic rate (RMR and MMR, respectively). Cylindrical acrylic respirometers (646 or 2040 ml) were connected to a water pump (Eheim, Deizisau, Germany) and tubing, creating a closed‐circuit recirculating loop to ensure continuous mixing throughout the respirometer (Clark *et al*., 2013). A fluorescent oxygen sensor T‐piece was included in the recirculating loop and connected to a Fibox 3 (PreSens, Regensburg, Germany) to noninvasively measure aquatic oxygen concentration (as % air saturation) continuously. A second circuit was attached to the respirometer to flush the respirometer with oxygenated water between measurement periods. This second circuit was closed during measurement periods.

To measure the RMR, fish were placed into respirometers with the recirculating pump and flush pump on for 30 min to recover from handling stress. Following this, the flush pump was turned off and the measurement cycle began. A maximum of three measurement cycles (10–30 min depending on fish size and % air saturation) were recorded and the lowest calculated RMR was used for analysis. The flush pump was turned on between measurement cycles to oxygenate the water. To measure the MMR, fish were placed into respirometers immediately after exhaustion using an exhaustive chase protocol. The exhaustive chase protocol consisted of chasing fish until exhaustion (exhaustion defined as inability to perform bursts of swimming) (Norin & Clark, 2016) in a temperature‐controlled water bath (Clayson, Narangba, Australia; water bath dimensions = L × W × H, 50 × 30.5 × 19 cm). Following this, aquatic oxygen concentrations were measured every minute for 15 min and the highest calculated MMR was used for analysis. $\dot{M}O_{2}$ (mg O_2_ h^−1^) was calculated using the equation:

$$\dot{M}O_{2}=-1\times {\mathrm{\Delta O}}_{2}\times V\times {\mathrm{\beta O}}_{2}$$

where ΔO_2_ is the rate of change of oxygen saturation within the respirometer (as % air saturation per hour), *V* is the volume of the respirometer minus the mass of the fish (assuming a density of 1 g ml^−1^) and βO_2_ is the solubility of oxygen in water at the given temperature (Cameron, 1986).

### 2.6 Data analyses

Analyses were performed using the statistical package R (R Core Team, 2013) within the Rstudio platform (version 0.98.1103). Linear mixed‐effects models were used to examine the effects of test and acclimation temperatures and acclimation time on each performance trait using the *lmerTest* package (Bates *et al*., 2015; Kuznetsova *et al*., 2017). *‘Treatment’* and *‘Week’* were treated as fixed factors for analysis of time course of exposure for swimming performance measures. *‘Treatment’* and *‘Test temperature’* were treated as fixed factors for analysis of final measurements of swimming performance and metabolic rate at the treatment temperature and the opposing temperature. *‘Fish ID’* and *‘Tank’* were included as random effects to account for multiple measures of the same individuals and from the same tanks throughout the experiment. *‘Total length’* and *‘Mass’* were included as a covariates for swimming performance and metabolic rate, respectively. Metabolic rate and growth data were log‐transformed to meet underlying model assumptions. The ‘step’ function in the *lmerTest* package was used for model selection and the ‘ranova’ function was used to determine if random effects were contributing to variation within the model where the ‘step’ function could not be used. The Tukey HSD *post hoc* test was used for least squared means comparisons to determine differences between treatments/test temperatures. Survival data were modelled using a Cox mixed effects model in the *coxme* (Therneau, 2020b) and *survival* (Therneau, 2020a; Therneau & Grambsch, 2000) packages and *‘Tank’* was included as a random effect. The ‘survdiff’ function in the *survival* package and the ‘glht’ function in the *multcomp* package (Hothorn *et al*., 2008) were used for *post hoc* comparisons between survival curves.

## 3 RESULTS

### 3.1 Growth

*M. peelii* increased significantly in mass and length over time (total length: *F*_4,213.02_ = 312.65, *P* < 0.001; mass: *F*_4,213.31_ = 291.9, *P* < 0.001; Table 1), but there was no significant effect of temperature treatment on growth rate (total length: *F*_3,7.95_ = 0.41, *P* = 0.749; mass: *F*_3,7.91_ = 0.53, *P* = 0.675)

**TABLE 1:** Total average length and body mass of a subset of juvenile *Maccullochella peeli* from each treatment at the beginning and end of the 8 week exposure period (*n* = 16 per treatment measured)

	Initial		Final	
Treatment	Total length (cm)	Mass (g)	Total length (cm)	Mass (g)
Gradual, 14°C	10.3 ± 0.7	14.8 ± 2.5	12.4 ± 0.6	22.1 ± 3.3
Intermediate, 14°C	10.1 ± 0.5	13.1 ± 2.0	12.0 ± 0.5	21.3 ± 2.4
Rapid, 14°C	10.5 ± 0.5	15.4 ± 2.8	12.4 ± 0.6	23.7 ± 3.8
Control, 24°C	10.5 ± 0.7	16.4 ± 3.3	14.5 ± 1.0	49.1 ± 10.1

*Note*: Data shown as mean ± s.e.

### 3.2 Swimming performance

**FIGURE 2:**
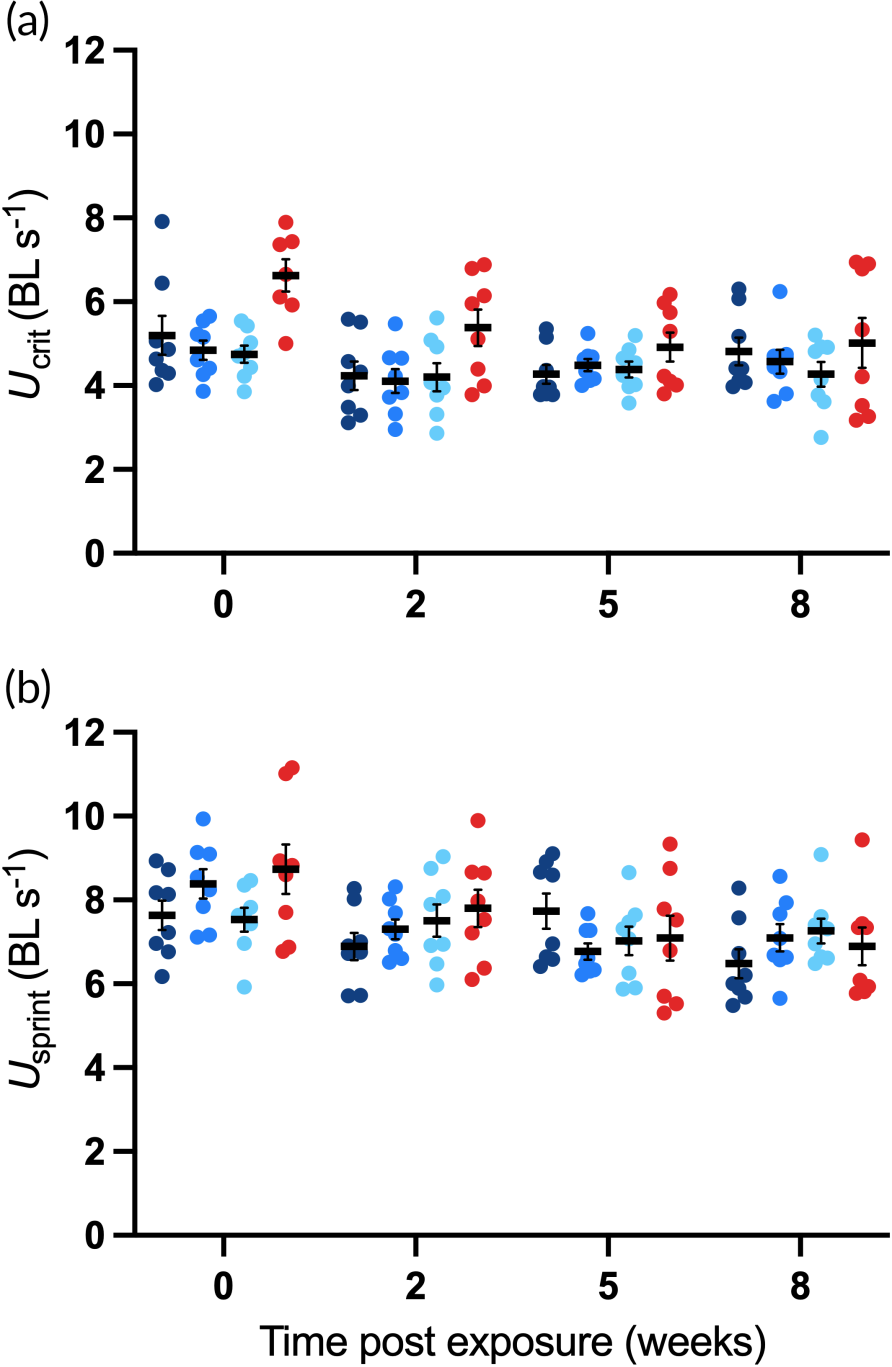
Critical (a) and sprint (b) swimming speed (body lengths s^−1^) of juvenile *Maccullochella peelii* in 14°C (gradual, intermediate and rapid exposure; blue symbols) or 24°C (red symbols) temperature treatments over exposure time (weeks). Body length corrected raw data are displayed and mean ± s.e. (*n* = 8 per treatment per time point). (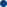) gradual exposure; (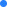) intermediate exposure; (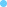) rapid exposure; (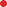) control

**FIGURE 3:**
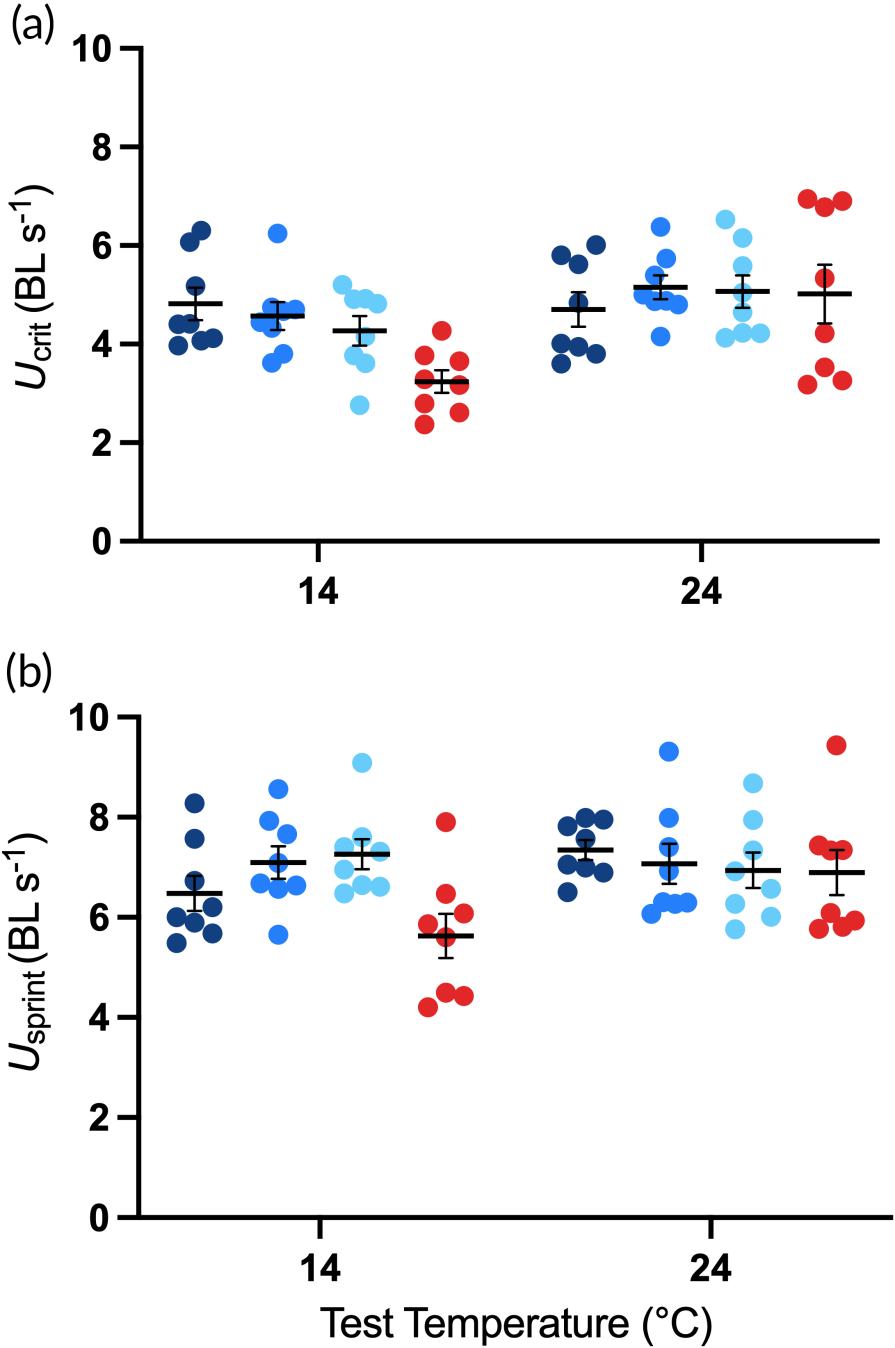
Effect of test temperature on critical (a) and sprint (b) swimming speed of juvenile *Maccullochella peelii*, acclimated to 14°C (gradual, intermediate and rapid exposure; blue symbols) or 24°C (red symbols) and tested acutely at both 14 and 24°C. Body length corrected raw data are displayed and mean ± s.e. (*n* = 8 per treatment per test temperature). (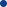) gradual exposure; (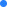) intermediate exposure; (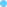) rapid exposure; (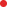) control

Prolonged exposure to 14°C resulted in an overall poorer performance for *U*_crit_ (*F*_1,65.59_ = 43.07, *P* < 0.001; Figure 2a) and *U*_sprint_ (*F*_3,365.5_ = 11.48, *P* < 0.001; Figure 2b) in juvenile *M. peelii*, regardless of the rate of temperature decrease (treatment). Following 8 weeks of chronic exposure to 14°C, there was no significant effect of treatment on *U*_crit_ in comparison to fish maintained at 24°C (*F*_3,21.30_ = 1.1, *P* = 0.358), but there was a significant main effect of test temperature (*F*_1,1148.43_ = 59.14, *P* < 0.001) and a significant interaction between treatment and test temperature (*F*_3,245.72_ = 12.65, *P* < 0.001; Figure 3a). Acute exposure to 14°C had a significant depressive effect on *U*_crit_ for fish maintained at 24°C (d.f. = 55, *t* = −8.986, *P* < 0.0001; Figure 3a). Fish acclimated to 14°C for 8 weeks and tested at 14°C had significantly higher *U*_crit_ than fish maintained at 24°C and acutely exposed to 14°C (gradual: d.f. = 55, *t* = 4.562, *P* = 0.0002; intermediate: d.f. = 55, *t* = 5.116, *P* < 0.0001; rapid: d.f. = 55, *t* = 3.429, *P* = 0.0062), suggesting some thermal compensation had occurred. However, the *U*_crit_ of fish maintained and tested at 24°C was still significantly higher than those maintained and tested at 14°C (gradual: d.f. = 55, *t* = −4.123, *P* = 0.003; intermediate: d.f. = 55, *t* = 3.715, *P* = 0.0105; rapid: d.f. = 55, *t* = −5.429, *P* < 0.0001), suggesting thermal compensation was only partial. For *U*_sprint_, there was no significant effect of treatment following 8 weeks of chronic exposure to 14°C in comparison to fish maintained at 24°C (*F*_3,63.1_ = 1.42, *P* = 0.247), but there was a significant main effect of test temperature (*F*_1,779.5_ = 17.55, *P* < 0.001) and a significant interaction between treatment and test temperature (*F*_3,287.9_ = 6.48, *P* < 0.001; Figure 3b). For fish maintained at 24°C, acute exposure to 14°C resulted in significantly lower *U*_sprint_ (d.f. = 55, *t* = −5.579, *P* < 0.0001; Figure 3b). *U*_sprint_ for fish maintained and tested at 24°C was not significantly higher than for fish maintained and tested at 14°C (gradual: d.f. = 55, *t* = −2.709, *P* = 0.1417; intermediate: d.f. = 55, *t* = −2.356, *P* = 0.2832; rapid: d.f. = 55, *t* = −0.943, *P* = 0.9802), suggesting complete thermal compensation.

### 3.3 Metabolic rate

**FIGURE 4:**
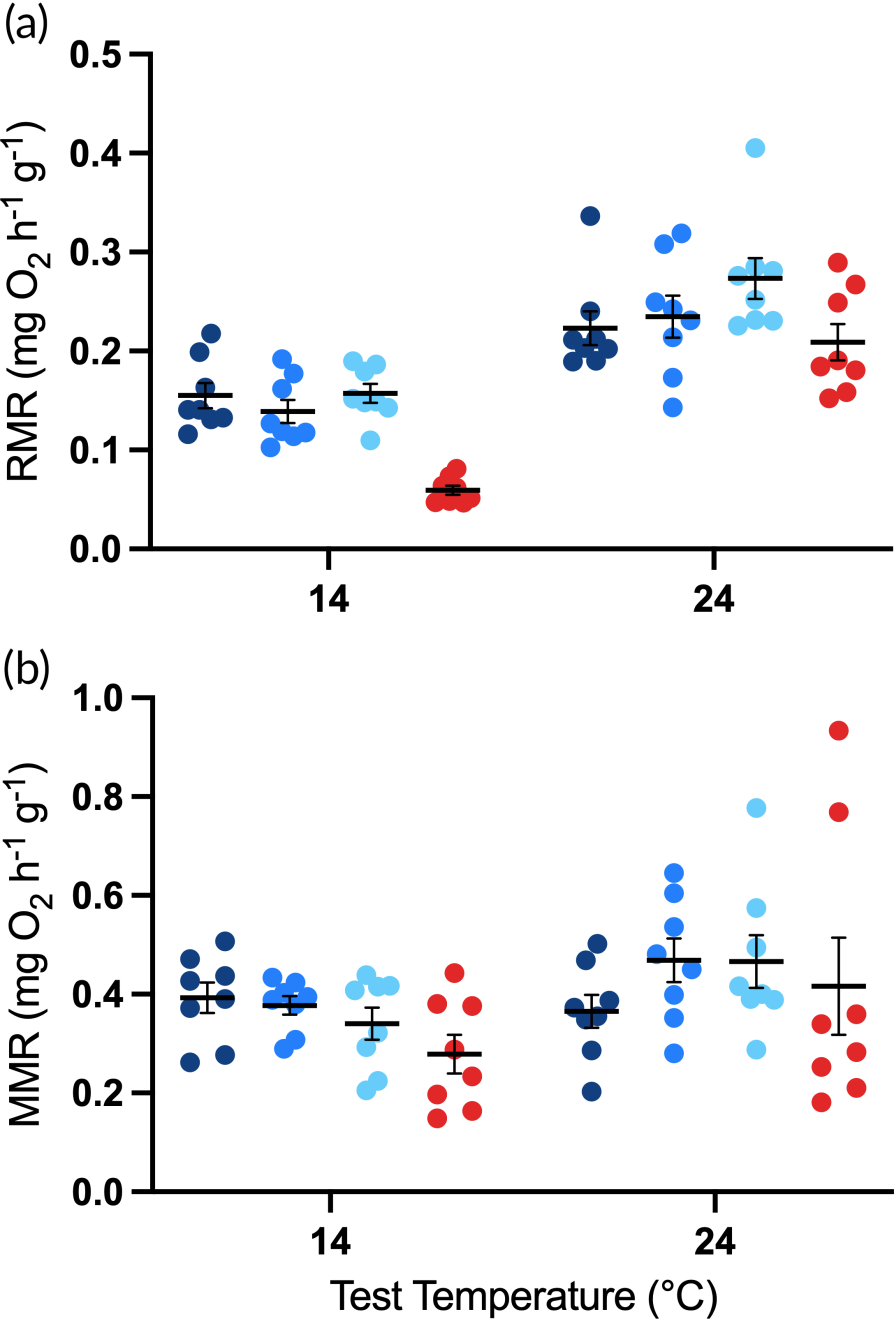
Effect of test temperature on routine (a) and maximum (b) oxygen consumption of juvenile *Maccullochella peelii*, acclimated to 14°C (gradual, intermediate and rapid exposure; blue symbols) or 24°C (red symbols) and tested acutely at both 14 and 24°C. Mass corrected raw data are displayed and mean ± s.e. (*n* = 8 per treatment per test temperature). (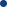) gradual exposure; (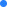) intermediate exposure; (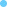) rapid exposure; (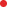) control

For fish maintained at 24°C, RMR and MMR were significantly decreased when acutely exposed to 14°C (RMR, Figure 4a: d.f. = 50.4, *t* = −2.91, *P* = 0.005; MMR, Figure 4b: d.f. = 48.71, *t* = −3.57, *P* = 0.0008). There was a significant main effect of test temperature on RMR (*F*_1,50.71_ = 43.15, *P* < 0.001) and a significant interaction between treatment and test temperature (*F*_3,50.37_ = 2.94, *P* = 0.042), but there was no significant main effect of treatment (*F*_3,8.73_ = 0.56, *P* = 0.654). RMR for fish maintained and tested at 24°C was not significantly higher than that for fish maintained and tested at 14°C (gradual: d.f. = 10.28, *t* = −0.028, *P* = 1; intermediate: d.f. = 10.62, *t* = −1.423, *P* = 0.8295; rapid: d.f. = 11.05, *t* = −0.647, *P* = 0.997), indicating complete thermal compensation. For MMR, there was a significant main effect of test temperature only (*F*_1,49.85_ = 11.14, *P* = 0.002). There was no significant difference in MMR for fish maintained and tested at 24°C compared to fish maintained and tested at 14°C (gradual: d.f. = 9.92, *t* = −1.409, *P* = 0.8346; intermediate: d.f. = 9.73, *t* = −0.953, *P* = 0.9718; rapid: d.f. = 9.19, *t* = −1.239, *P* = 0.9007), again indicating complete thermal compensation.

### 3.4 Survival

**FIGURE 5:**
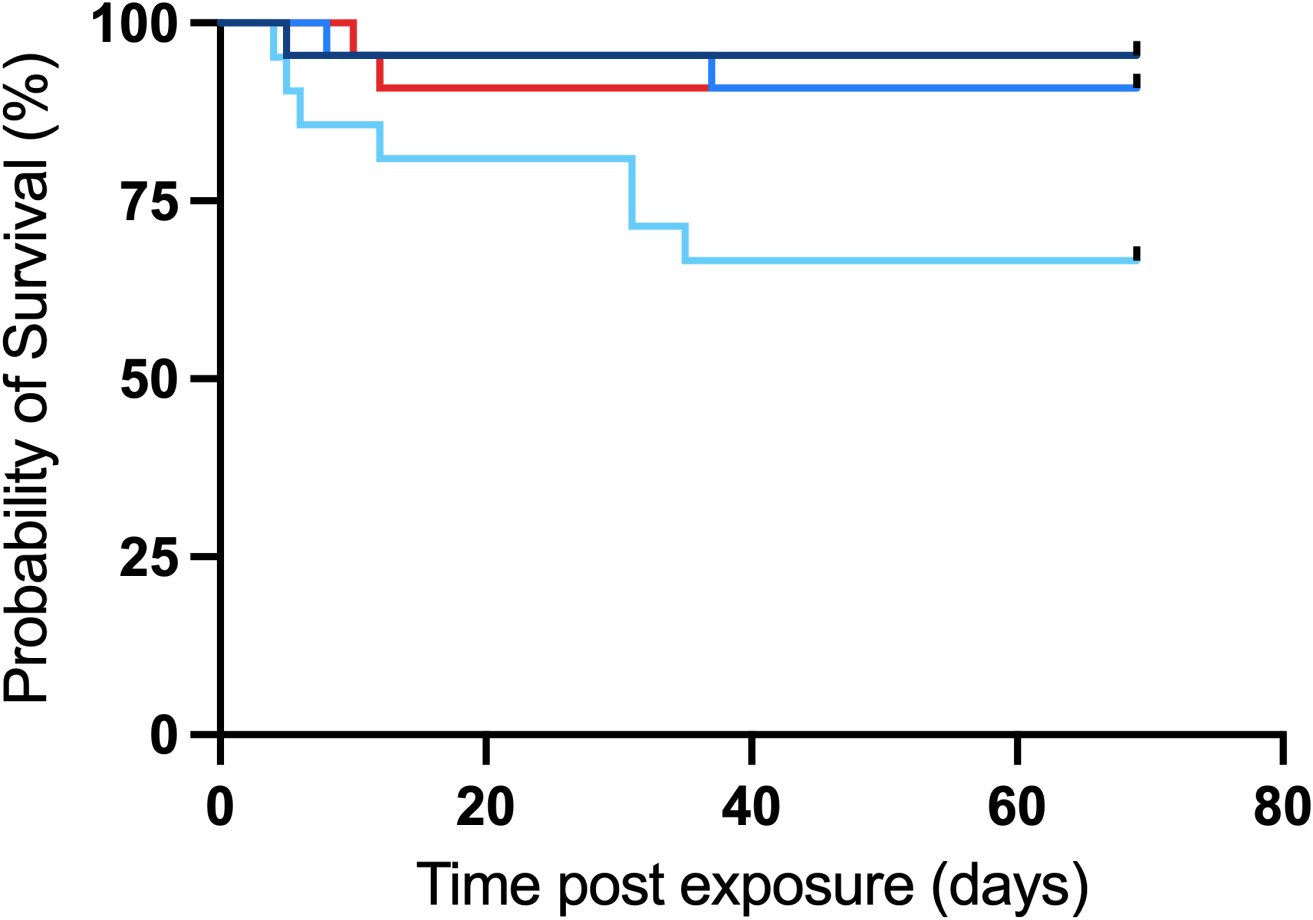
Effect of treatment on percentage survival of juvenile *Maccullochella peelii* (*n* = 21 for rapid exposure treatment and *n* = 22 for all other treatments) during 69 days of chronic exposure to 14°C (gradual, intermediate and rapid exposure; blue lines) or 24°C (red line). (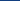) gradual exposure; (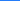) intermediate exposure; (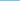) rapid exposure; (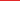) control

Treatment had a significant overall effect on survival (d.f. = 3, *P* = 0.027; Figure 5). However, *post hoc* analyses revealed no significant differences between treatments.

## 4 DISCUSSION

Acute reductions in water temperature associated with unseasonably cold water releases from thermally stratified dams can have significant effects on the physiological function of aquatic ectotherms in the downstream reaches of the waterway (Astles *et al*., 2003; Michie *et al*., 2020b; Parisi *et al*., 2020). However, managing the speed of the thermal change downstream by controlling water release rates could help to ameliorate the impacts of the overall thermal change by allowing physiological compensation (thermal acclimation). We tested this hypothesis in juvenile *M. peelii*, and although acute exposure to 14°C resulted in decreased swimming performance and metabolic rate measures, juvenile *M. peelii* did demonstrate a capacity to compensate for the depressive effects of low temperature over time. However, contrary to our hypotheses, there were no differences in the acclimation responses of fish from the cold treatments, irrespective of initial cold water exposure rates. This study has implications for the management of dam releases and indicates that slowing the rate of temperature decline to rates tested here may not be a successful mitigation strategy for CWP effects on *M. peelii*.

Thermal acclimation capacity can be influenced by rate of temperature change and amount of thermal variability during exposure (Nilsson‐Örtman & Johansson, 2017; Schulte *et al*., 2011). While we did not find an effect of the rate of temperature change on acclimation capacity in our study, it is possible that other physiological traits not measured here may have responded differently to these rates. In other organisms (*e.g*., springtail species (Allen *et al*., 2016), *Drosophila melanogaster* and *Linepithema humile* (Chown *et al*., 2009), and *Coenagrion pulchellum* and *Coenagrion armatum* (Nilsson‐Örtman & Johansson, 2017)), the rate of temperature exposure does impact the acclimation capacity of other thermally sensitive traits. For example, rate of temperature change is particularly important for experimental investigations of critical thermal limits (Beitinger *et al*., 2000), where studies have evidenced increases and decreases in critical thermal limits with variations in experimental rates of temperature change (*e.g*., Allen *et al*., 2016, 2012; Becker & Genoway, 1979). Additionally, Schulte *et al*. (2011) highlighted that exposure to realistic thermal conditions that mirror natural thermal regimes as opposed to constant treatment temperatures (as often maintained in laboratory experiments) can result in rapid changes to thermal phenotypes.

Although there were no differences in acclimation responses between cold treatments, *M. peelii* demonstrated differences in acclimation responses across physiological metrics, indicating that the capacity for thermal plasticity can differ between metrics. We previously demonstrated a similar effect in *B. bidyanus* whereby fish acutely exposed to a 10°C water temperature reduction showed complete compensation of metabolic rate but only partial compensation of *U*_crit_ after 10 weeks of exposure (Parisi *et al*., 2020). Different acclimation capacities between traits may be due to differing underlying physiological mechanisms. For example, differences in performance between swimming styles with temperature is reflective of the thermal sensitivity of different muscle fibre types and metabolism powering that performance (Guderley & Blier, 1988). Furthermore, the contractile properties of muscle fibres, enzyme activity, fibre recruitment order and metabolic properties all undergo changes during thermal acclimation (Guderley & Blier, 1988). Specifically, aerobic enzymes (*e.g*., citrate synthase and cytochrome *c* oxidase) in muscle tissue can increase in activity with low temperatures, while anaerobic enzymes (*e.g*., lactate dehydrogenase) decrease (Guderley & Blier, 1988; Parisi *et al*., 2020). Therefore, swimming styles that rely on aerobic pathways may have higher acclimation capacities than those that predominantly rely on anaerobic metabolism. Due to differences in thermal acclimation capacities between traits, it is essential to measure a number of physiological metrics to gain a better understanding of the impacts on fish.

Acclimation responses can also vary with body size of individuals and length of exposure time to acclimation conditions (acclimation duration) (Rohr *et al*., 2018). Although our study focused on juvenile *M. peelii*, this species can vary in body size by over three orders of magnitude. Had we conducted the same experiment on larval or adult life stages, would we have found the same acclimation responses? Acclimation is time‐sensitive, consequently, acclimation capacities can be underestimated where experimental acclimation durations are short because individuals may not have been given sufficient time to completely acclimate to conditions being tested (Rohr *et al*., 2018). Additionally, smaller‐bodied organisms undergo acclimation faster relative to larger‐bodied organisms and they can be more thermally sensitive, as they heat and cool faster (Rohr *et al*., 2018). This may have important implications for acclimation responses across life‐history stages (Klockmann *et al*., 2017). Management decisions based solely on data for one body size (or life history stage) may not be reflective of all life‐history stages. Furthermore, current experimental CWP studies focus largely on juvenile fishes (*e.g*., Astles *et al*., 2003; Parisi *et al*., 2020), which highlights the need for future research to be conducted on larval and adult fish to better understand the impacts of CWP on these life‐history stages.

In addition to the sublethal impacts evidenced here, lethal impacts can also occur for species downstream of cold water releases. In a study by Astles *et al*. (2003), *B. bidyanus* survival was lower than 50% in cold water channels, in comparison to warm‐water channels representative of summer temperatures in the MDB. Lethal effects could be due to the cascading physiological responses associated with cold shock stress from rapid decreases in temperature (Donaldson *et al*., 2008). This highlights the importance of the rate and magnitude of temperature decreases with cold water releases, as current dam‐release practices allow temperatures to rapidly plummet by up to 16°C, resulting in acute lethal and sublethal effects on downstream fish populations (Lugg & Copeland, 2014). However, as seen here, thermal plasticity and the underlying mechanisms of compensatory responses can aid in offsetting sublethal effects on performance in some fish species.

The results presented here and in previous studies (Astles *et al*., 2003; Lugg & Copeland, 2014; Parisi *et al*., 2020) demonstrate that the impacts of CWP‐like thermal regimes can be widespread among physiological and whole‐organism traits in fish, contributing to the effects that barriers have on the fragmentation of waterways. Although phenotypic plasticity can allow some species to overcome the negative effects of cold stress from CWP events, our findings suggest that it may not be sufficient to completely mitigate the impacts of CWP on *M. peelii*, especially in circumstances where cold water exposure has lethal effects and/or compensation does not occur rapidly. This has significant management implications for freshwater communities downstream of large impoundments. First and foremost, it is critical that water temperatures remain within optimal thermal ranges for both performance and reproduction in the breeding season to promote the success and recovery of native fish species in fragmented and thermally polluted waterways. To achieve this, thermal ranges need to be identified for target species at various life stages and from those data temperature targets within thermal ranges can be set for dam releases. Where possible, release windows should occur during noncritical periods (*i.e*., outside of breeding season) in cases where releases are going to rapidly reduce downstream temperatures. Another strategy is to raise the temperature (and/or reduce thermal stratification) of water released from thermally stratified dams, but achieving this is an ongoing challenge as feasibility and logistics have prevented many current mitigation measures pertaining to this strategy (Michie *et al*., 2020a; Sherman, 2000).

It is important to note that we demonstrate how physiological data can provide evidence for future management strategies by measuring physiological responses to CWP‐like temperature reductions ‘played back’ in the laboratory setting. Using physiological data to inform management and/or policy decisions is becoming increasingly recognized as a tangible strategy for addressing conservation issues (Cooke *et al*., 2013; Madliger *et al*., 2020; Madliger & Love, 2015). Conservation physiology is particularly well‐positioned to identify problems and solutions surrounding fish passage (Cramp *et al*., 2020). Laboratory‐based swimming performance estimates (*U*_crit_ and *U*_sprint_) are critical in providing insight into the capacity of free‐swimming fish to perform critical behaviours in the field (Peake, 2008), especially when the capacity to measure and interpret these behaviours in the field can be limited by high cost, small sample sizes and specialized training requirements (Cooke *et al*., 2004). The management of CWP is a complex task, but physiological data has the potential to provide crucial insights into the function of fishes that can inform policy and the management of freshwater ecosystems downstream of large, thermally stratified dam impoundments.

## AUTHOR CONTRIBUTIONS

M.A.P: conceptualization (lead), investigation (lead), methodology (lead), formal analysis (lead), writing – original draft (lead), writing – review and editing (equal). C.E.F: conceptualization (supporting), investigation (supporting), methodology (supporting), writing – review and editing (equal), funding acquisition (equal). R.L.C: conceptualization (supporting), investigation (supporting), methodology (supporting), writing – review and editing (equal), funding acquisition (equal).

## Data Availability

Data are available online on UQ eSpace at https://doi.org/10.14264/e283cf8.
